# Seroprevalence of Q fever (*Coxiella burnetii)* in sheep in the Kwahu West municipality, Eastern Region, Ghana

**DOI:** 10.1016/j.heliyon.2024.e33009

**Published:** 2024-06-13

**Authors:** Richard Kwamena Abbiw, Gloria Ivy Mensah, Delphina A.M. Adabie-Gomez, Kweku Asare-Dompreh, Stephanie Clement-Owusu, Vida Yirenkyiwaa Adjei, Shirley Victoria Simpson, Mustapha Abubakar Ahmed, Sherry A.M. Johnson

**Affiliations:** a School of Veterinary Medicine, University of Ghana, Legon, Accra, Ghana; b Noguchi Memorial Institute for Medical Research, University of Ghana, Legon, Accra, Ghana; c West African Centre for Cell Biology of Infectious Pathogens (WACCBIP), University of Ghana

**Keywords:** Q fever, Coxiella burnetii, Abortion, One-health, Ghana

## Abstract

Query fever, also known as Q fever, is a zoonotic disease caused by *Coxiella burnetii*. It is a cause of abortion in livestock and presents as a febrile illness in humans. A correlation between the incidence of the disease in humans and abortion in goats and sheep farms has been reported in countries such as the Netherlands and Australia. In Ghana, the occurrence of Q fever in both livestock and humans has not been fully explored. This study sought to determine the seroprevalence of Q fever in livestock in Nkawkaw, in the Eastern Region of Ghana. Sera obtained from 92 sheep from 12 farms were tested using the indirect multi-species ELISA for the detection of anti-*Coxiella burnetii* antibodies. Animal demographics, farms’ proximity to human settlement and history of abortion in relation to the Q fever status were assessed.

The overall prevalence of Q fever was 13.0 % [95 % CI 6.9–21.6] (12/92). Both sexes were equally affected, with a sex-specific prevalence of 13.0 % each. The farm-specific prevalence was 50 %. Abortions were reported on eight (8) of the 12 farms, and all farms were located less than 200 m from human habitation. Only proximity of farm to human settlement showed statistical significance. Q fever is prevalent in Nkawkaw and requires the attention of both animal and health authorities, using the One- Health approach to nip any future epidemics in its bud.

## 1 Introduction

Q Fever is a bacterial zoonotic disease caused by *Coxiella burnetii* [1]. The disease, reported globally except in French Polynesia, Antarctica, and New Zealand [1,^2^], presents epidemiologically as sporadic, endemic, hyperendemic, or epidemic [3]. Abattoir workers, animal health workers and livestock farmers are among the people a risk of exposure to infection [4]. The zoonotic disease is considered ubiquitous, underreported, under-diagnosed and neglected, especially in resource-poor countries [5]. The difficulty associated with the diagnosis of the infection in the past [3], the excessive attention and resources demanded by the sporadic distribution of Q fever cases [6], and the attribution of most cases of Q fever to other febrile or flu-like diseases in humans [7] are some of the reasons for these observations.

However, recent global events, including (i) the 2007–2010 outbreak of Q fever in the Netherlands [8]; (ii) the increase in research into the contribution of Q fever to Fever of Unknown Origin (FUO) [9]; (iii) the recognition of the role of *C. burnetii* as a cause of endocarditis [3]; (iv) the acquisition of a Category B Bioterrorism Weapon status [3]; and (v) the increase in the general knowledge about *C. burnetii* [10] have increased the attention given to the bacterium. Despite these, Q fever has received little to no attention in Africa and its impact on human and animal health is poorly estimated.

Q fever is not a reportable disease for animals or humans [11]. The disease, although not prioritized, was considered one of the zoonotic diseases of one-health importance in Ghana [12]. A handful of investigations have been conducted regarding the occurrence of the disease in ruminants and high-risk individuals in Ghana (Table 1). Kobbe et al. initially estimated the risk of the disease in children and found it to be higher among children of illiterate mothers [13]. They observed that, contrary to literature [14], dairy played no role in the transmission of the bacteria in Ghana. The association of the transmission of Q fever and individuals in contact with ruminants or ruminants’ excretions and afterbirths was also not explored. Yeboah [15] explored the seroprevalence of Q fever among febrile patients and concluded Q fever as a cause for hospitalization among such patients. Occupation and employment were identified as two main factors that increased the risk of acquiring Q fever [15]. In a study by Johnson et al. an increased prevalence of Q fever in sheep compared to cattle and goats was reported [16]. Contrary to Johnson et al., Folitse et al. showed a higher prevalence in goats than sheep [16,17]. Assessment of the knowledge, attitudes and practices of at-risk individuals identified improper disposal of afterbirth material, and proximity of ruminant farms to human settlement as major risk factors for the occurrence of Q fever [16]. However, none of the studies conducted in Ghana has demonstrated a statistical significance to sex, age or any other parameter both in humans and animals [13,[15], [16], [17], [18]].

**Table 1:** Seroprevalence studies on Q fever in domestic ruminants and humans in Ghana.

Authors	Year	Location	Prevalence
Kobbe et al. [13]	2008	9 rural areas in the Ashanti Region	16.9 % children 8.9 % adults
Adu-Addai et al. [18]	2012	Apolonia, Coastal Ghana	17.7 % cattle
Yeboah [15]	2016	Accra and Sekondi Tarkoradi	16.2 % humans
Johnson et al. [16]	2019	Tongu	28.4 % sheep 21.7 % cattle 10 % goats
Folitse et al. [17]	2020	Kumasi abattoir	22.3 % overall 28.5 % goats 16.6 % sheep

The objective of this study was to determine the occurrence of Q fever in sheep in Nkawkaw in the Eastern Region of Ghana to contribute to the growing evidence of the wide distribution (and near endemicity) of Q fever in Ghana.

## 2 Method

### 2.1 Study area

**Fig. 1:**
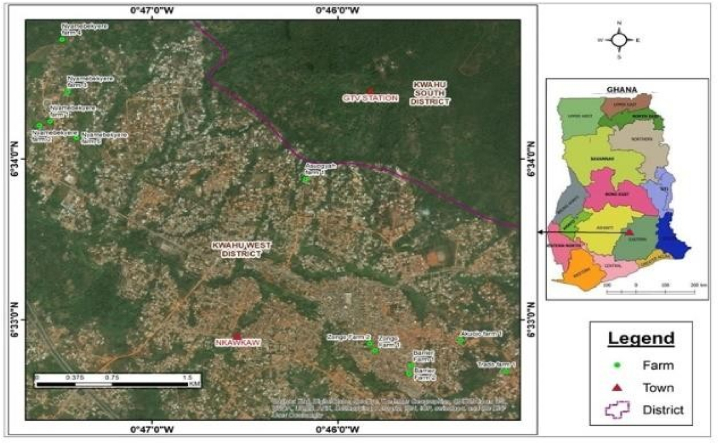
Map of Ghana showing study sites in Nkawkaw, Ghana.

The study was performed in Nkawkaw, in the Kwahu West Municipality of the Eastern Region of Ghana (Fig. 1). The main occupation in the municipality is crop and animal farming. Only poultry is done on a commercial scale [19]. Nkawkaw was selected because of the absence of data on the occurrence of Q fever in the municipality. The ubiquity of small ruminants, kept for subsistence and preferably via semi-intensive systems, made Nkawkaw a prime candidate for the study.

#### 2.1.1 Sample size and sampling

The participating farms met three predetermined inclusion criteria. The farm had to be registered with the veterinary service offices, receive regular visits from veterinary professionals, and have more than 5 sheep above 3 months old. Twelve farms were then randomly selected from shortlisted farms provided by the Veterinary Services Office in Nkawkaw. Sheep, above three months of age, were then randomly selected and sampled from the flocks on the selected farms.

Eq.1 $$\text{The}\;\text{sample}\;\text{size}\;\text{was}\;\text{calculated}\;\text{using}\;\text{the}\;\text{formula}:\left(n=\frac{Z^{2}p\left(1-p\right)}{d^{2}}\right)$$

With a prevalence [20] (p) of 28.4 % in sheep [16], a precision (d) of 10 %, and Z at an alpha (α) of 5 %, the minimum sample size was calculated to be 78 sheep. However, a total of 92 sheep were sampled.

### 2.2 Sample collection, transportation, and storage

Three millilitres (3 ml) of blood was collected from the jugular vein of each sheep and dispensed into an appropriately labelled serum separator tube. The tubes were made to stand in tube holders and transported in Coleman box (4–8 °C) to the Small Animal Teaching Hospital Laboratory, of the School of Veterinary Medicine, University of Ghana, Legon, Accra.

### 2.3 Laboratory analysis: ELISA using in-vitro indirect ELISA test Id Screen®

The process involved three main stages: serum harvesting and storage, preparation of reagents and samples, and the testing procedure. These steps were then followed by a validation test and the interpretation of the results. An approved Standard of Operation for testing for ovine and caprine Q fever in sheep and goats using the ID SCREEN ® FQS-MS ver 0514 GB Q- Fever ELISA Kit ELISA (France**)** [21,22]**,** adapted from the manufacturer was followed to detect antibodies to Phase I and Phase II strains.

Briefly, serum was extracted, by centrifuging the samples at 4000 rpm for 3 min and dispensed into 1.5 ml Eppendorf tubes with corresponding labels. All reagents and samples were prepared and allowed to attain room temperature (21 °C ± 5 °C) before use. To avoid differences in incubation times between specimens, a 96-well plate containing the test and control specimens was prepared before transferring them into an ELISA microplate using a multichannel pipette. The test was then carried out using standard protocol. To validate the test, the Optical Density (OD) of the Positive Control (OD_PC_) and the ratio of the mean value of the Positive Control OD (OD_PC_) and Negative Control OD (OD_NC_) were compared to threshold values of 0.350 and 3, respectively, set by the manufacturer. The acquired values for the OD_PC_ and OD_PC_/OD_NC_ were 1.393 and 28.722, respectively. Since these values were greater than the threshold values set by the manufacturer, the test was deemed valid, and interpretation of the result was done.

For each sample, the S/P percentage (S/P %) was calculated using the formula below (Eq. (2)).

Eq. 2 $$\frac{S}{P}\%=\frac{\text{ODsample}\;-\;\text{ODNC}\;}{{\text{OD}}_{\text{PC}}-\;\text{ODNC}}*100$$

Based on the calculated S/P % values, the samples were assigned strong positive (S/P % > 80 %), positive (50 % < S/P % ≤ 80 %), negative (≤40 %), or doubtful (40 % < S/P % ≤ 50 %).

### 2.4 Data analysis

The variables measured were categorised into three broad headings: variables concerning the sheep; variables concerning the farm; and variables concerning laboratory analysis. The variables concerning the sheep to be sampled were age, sex, history of abortion, and the health status at the time of sampling. The variables related to the farm were the proximity of the farm to the nearest human settlement, the farming system, the estimated average number of abortions recorded on the farm per year, how birth materials are discarded, sources of replacement animals, sources of labour, and the common diseases experienced by sheep on the farm. The variables concerning the sheep and farm were captured in a questionnaire**.**

All data were inputted into IBM SPSS 25, and descriptive statistics such as frequencies and percentages were obtained and compared. Chi-square was used to compare categorical variables. The analysis was considered statistically significant if p < 0.05.

## 3 Results

### 3.1 Farm Information

**Fig. 2:**
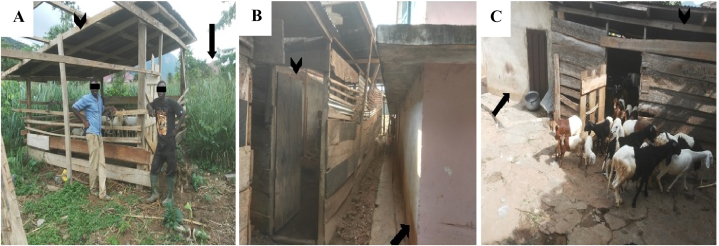
The distance between human residence and sheep pens. Arrowhead shows sheep pen and arrows show closest human residence. (A) a farm located about 100 m from a house. (B) a farm less than 10 m from the nearest house and (C) a farm within the walled compound of a house.

All the farms sampled were small scale or backyard small ruminant farms with farm size ranging from 8 to 35 animals. The farms had existed for an average of 10 years, with a minimum and maximum of 3 and 39 years, respectively. A third of the farms (4/12) practiced semi-intensive farming systems. All 12 farms were situated less than 200 m from the nearest human settlement. Eight (66.7 %) farms were less than 10 m away from the nearest human habitation (Fig. 2). The farms were either attached to the house or within walled human residence.

The sheep sampled were mainly females (75.0 %). The ages of the sheep ranged from 4 to 240 months with a median of 36 months. They appeared healthy with only 6.5 % exhibiting clinical signs such as conjunctivitis (28.6 %), paleness of mucous membranes (14.3 %) and diarrhoea (14.3 %). Abortion was reported on 7 (7.6 %) of the farms 6 months prior to sampling and there was no association between abortion and Q fever on the farms (p = 0.28). However, an ewe that aborted three days prior to the sampling tested positive. Communal grazing was common, with sheep from semi-intensive farms feeding in the same areas where pasture for intensive farms was sourced.

### 3.2 Prevalence of Q fever

The overall prevalence of Q fever was 13.0 % [95 % CI 6.9–21.6]. From Table 2, sex and age-group were not found to be associated with high seroprevalence. Only the proximity of farm to human settlement (p = 0.03) was significant.

**Table 2:** Prevalence of Q fever and risk factors in the Kwahu West municipality, Ghana.

Variable	Total sampled	Prevalence (%)	P value
Overall	92	13.04	
Sex			
**Female**	69	13.04	0.26
**Male**	23	13.04	
Age-group (months)			
**≤12**	6	0.00	
**13–47**	61	13.11	0.80
**>47**	25	16.00	
Distance from human settlement			
**10 m**	51	5.88	
**50 m**	37	24.32	0.03
**100 m**	4	0.00	

#### 3.2.1 Seroprevalence

**Fig. 3:**
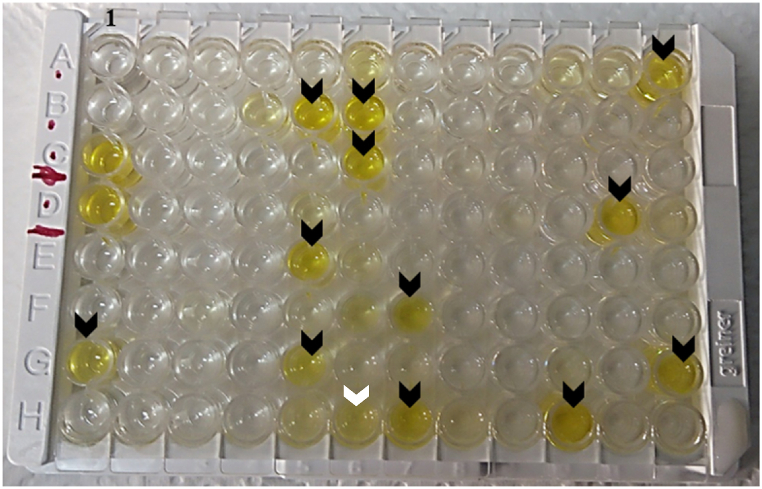
An ELISA plate displaying results. The black arrowheads represent positive results while the white arrowhead (H6) represent doubtful result. A1 and B1 are the negative controls while C1 and D1 are the positive controls.

Out of the 92 samples tested for *C. burnetii*, 1 (1.09 %) was doubtful, 79 (85.87 %) negative, 4 (4.35 %) positive, and 8 (8.70 %) strong positive (Fig. 3). In all, 12 (13.04 %) of the samples tested were positive for *C. burnetii*. Therefore, the prevalence of *C. burnetii* in Nkawkaw was 13.04 %.

##### 3.2.1.1 Characteristics and management of the farms

None of the farmers knew about Q fever in humans or animals. All farms that reported abortion (8/12: 66.7 %) attributed it to other sources, including plant toxins and accidental herbicide poisoning. They further indicated that afterbirth or abortion materials (birth materials) were either tied in plastic rubber and disposed of in communal bins (2/8: 25.0 %) or tossed/left in nearby bushes (6/8: 75 %) with two farms occasionally burying the material in a shallow hole (<30 cm deep). The distance between the farms and the bushes where the *birth* materials were discarded ranged from 100 to 300 m.

Most farmers purchased their sheep from nomadic herdsmen (83.3 %) or from other farmers (41.7 %). The new stock was not screened for any infections or quarantined before being added to the flock. All semi-intensive farms practiced communal grazing, while all farms sourced their pasture from the same natural pastures within and on the outskirts of Nkawkaw.

Slaughtering of sheep was done in abattoirs (16.7 %) or on the farm (75.0 %). One farmer (8.3 %), who kept the sheep as pets did not slaughter the sheep but instead gifted or sold only live sheep. All participating farms confirmed on-farm sales as the major means of disposing of the sheep. That is, interested buyers walk into the farm and select the preferred sheep.

**Fig. 4:**
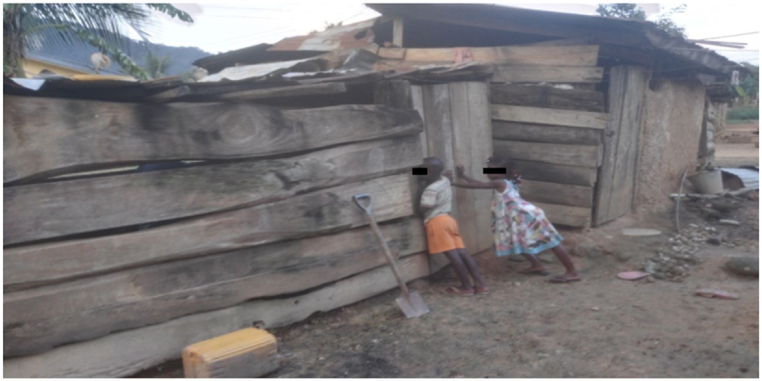
Children helping in tending to the sheep.

The farms were closer to their houses (less than 200 m) as the sheep were kept mostly for subsistence, and the family (including children; Fig. 4) provided labour and security. The farms were predominantly multispecies (58.3 %). Goats, poultry, and cattle were kept together with the sheep on 41.7 %, 16.7 % and 25.0 % on the participating farms respectively.

## 4 Discussion

This study sought to determine the seroprevalence of Q fever in Nkawkaw, in the Kwahu West Municipality, Eastern Region, Ghana. The prevalence of 13 % obtained in this study is important given that this disease hitherto had not been reported from the Municipality and considered a low priority disease in Ghana, both for animals and humans. The implication of this finding is that the bacteria may have been circulating among livestock and possibly humans without being identified.

The observed seroprevalence is within the estimated range for small ruminants in Africa (1.0–33.0 %) [4] but slightly lower than the global average in sheep of 15 % [23].

The seroprevalence was lower in this study as compared to prevalence of 28.4 % reported by Johnson et al. for sheep in the Tongu area in the Volta region of Ghana [16]. The relatively higher prevalence, akin to those obtained in El Minya Governorate in Egypt (25.7 %) [24], Iran (27.5 %) [25], and Gran Canaria Island in Spain (31.7 %) [26], could be attributed to high small ruminant densities and trading [16,27]. However, Northern La Cote d’Ivoire (9.4 %) [27] and Togo (15.0 %) [28] presented seroprevalences about the same as those obtained in this study. Based on the presence of Q fever in Nkawkaw, Tongu [15], selected areas in Ashanti region [12,16] and some Coastal towns [14,17], and the neighbouring countries bordering Ghana [24,25], this study agrees with the projection made by Johnson et al. that the disease might be widespread in Ghana [16]. It also justifies, to some degree albeit not prioritized, the inclusion of Q fever as a zoonotic of one health importance in Ghana [11]. Further studies in other areas of Ghana such as the Oti, Bono, and Ahafo regions where small ruminant rearing is vibrant is warranted.

There appeared to be a pattern of infection among the various variables analysed. For instance, infection is greater in animals younger than 48 months than above 48 months or males than females. But these were of no statistical significance. In terms of age, sex, pregnancy, and abortion status comparable claims were drawn by Kanouté et al. [27], Edalati-Shokat et al. [25] and Abushahba et al. [24]. That is, all groups (age, sex, etc.) are relatively at equal risk of acquiring the infection. Van den Brom et al. and Muema et al. assert that there is a significant positive correlation between the age of sheep and the seroprevalence of *C. burnetii* with peaks seen between 2 and 3 years [29,30]. Muema et al. attributed this to the practice of communal grazing, which increases the frequency of contact between different herds [30]. Again, while both (communal grazing and increased seropositivity among sheep between 2 and 3 years) were observed, there were no significance associated with these parameters.

It is unclear how a higher seroprevalence was reported in females than males. However, it is accepted that the differences in seroprevalence could be attributed to high variation in both sexes, influenced by preferential selection of females over males on farms [24], as observed in this study. Cantas et al., and Porter et al., on the other hand, suggest the role of oestrogen in immunosuppression and the perceived increase in female susceptibility to infection compared to males as rationale justifications [14,31].

The sources and spread of the bacteria in the herd could be attributed to the following reasons: (1) the purchase of already infected animals from the nomadic herds; (2) indiscriminate disposal of birth materials; (3) the grazing of animals of different flocks on the same pasture; (4) multispecies farming; and (5) the possibility of *C. burnetii* being a resident of the community since antiquity. The latter can be justified by the perceived global distribution of the bacteria [2].

The close relationship between nomadic shepherds and their animals as well as other farms as they traverse several countries makes them and their animals potential carriers of many infections, including Q fever [28,30,32]. Furthermore, the complete absence of screening for Q fever and most Neglected Tropical Diseases (NTDs) in these herdsmen and their animals as they enter and leave Ghana denies both them and Ghana an opportunity to manage any possible diseases they and their animals might carry. They and their animals could therefore be considered a potential source of *C. burnetii* for the 83.3 % farms that claimed to purchase animals from the herdsmen. This is, however, not conclusive, as the sources of animals sampled were not traced. It will be expedient for both the Veterinary Service Directorate (VSD) and Ghana Health Services (GHS) to consider implementing and/or enforcing measures aimed at screening, quarantining and treating herdsmen and their animals entering and leaving the country.

The fact that most of the bacteria are shed through afterbirth and abortion materials [3] makes the method of disposal of afterbirth and abortion materials very pertinent. Thus, regardless of the statistical significance associated with the relationship between infection and abortion status or infection and mode of disposal of peripartum materials, it is of clinical relevance. The indiscriminate disposal of birth materials could serve as a source of infection for other animals and humans living in close proximity to infected farms [16]. Animals and humans that directly encounter these materials or live within a 3 km radius are at risk of getting infected [33]. That an ewe that aborted three days prior to sampling was positive for *C. burnetii* is suggestive of the ewe being a potential source of spread of the infection to neighbouring animals and people. This might be demonstrative of how palpable the risk of Q fever is in the sampled communities.

The role of other ruminants as reservoirs of *C. burnetii* has been established [29]. Hence, the presence of other animals on the farms could be a source of infection for the sheep, if not otherwise or both ways. This can be associated with the deferential infection rates of different ruminants. For instance, there are evidences to support the increased rate of infection in goats compared to sheep [8,24,25,27,34,35]. The alternate is argued [16,36]. While the differential infection rate or carrier status amongst various animal species kept on the farms was not examined in this study, there is enough pre-existing evidence from literature to suggest it as a plausible risk of infection [16,37].

Communal grazing of animals poses a great threat to uninfected animals. This system of grazing increases the contact between animals of different herds. Hence, the uninfected animals could acquire the infection from the body discharges of infected animals and afterbirth materials left in the field to decay. Since intensive farms relied on the same pastures used by other semi-intensive farms, the bacteria could have been introduced on their farms by the cut pastures brought to the farm. For intensive farms, the risk could be reduced by treating the pasture before feeding it to their flock.

Even though this study did not include humans, the presence of infected animals in all sampled communities poses a palpable risk. Some of the potential risk factors, including interactions with nomadic herds (especially involved in transhumance) and improper disposal of birthing materials identified for animals in this study also apply to humans. All humans living close to ruminant farms are at risk. While it might be improbable to convince all farmers to relocate their farms, these farmers can be educated on the steps to reduce the likelihood of infection. This includes, and at the very least, proper disposal of afterbirth materials (for example deep burial or incineration of these waste). The farmers can also be encouraged to inform their physicians about their profession and incidences of abortion on the farm to help physicians consider certain zoonoses, including Q fever, as differentials when dealing with febrile illnesses in farmers. The VSD and GHS should collaborate and champion public education on zoonotic diseases, including Q fever. They can also incorporate zoonoses and one health approaches into their continuous professional development (CPDs) courses to help physicians and veterinarians become conversant with these diseases.

## 5 Limitations

The study was limited to only sheep. This presents a narrow view and makes addressing of inter-species and human risk rather hypothetical. Future studies should consider sampling both humans and other domestic animal species. It can also include other areas where human and animal interactions are inevitable like abattoirs and checkpoints for animal transports. A nationwide study can also be conducted to estimate the true prevalence and risk of the disease or bacterium.

Further studies of other possible causes of abortion in the study area can contribute to the determination of the actual contribution of Q fever to abortions. Finally, future studies will also benefit from the use of other molecular techniques to demonstrate the pathogen.

## 6 Conclusion

Q fever was found in sheep distributed in all the communities sampled in Nkawkaw in the Kwahu West Municipality of the Eastern Region of Ghana. Proximity of human settlement was found to be associated with the prevalence. The major risk factor was the proximity to human residence. Further studies into the seroprevalence of *C. burnetii* should be conducted in humans and other domestic ruminants to identify and analyse the risk of humans acquiring the pathogen from animals. The study should be conducted in other parts of the country to determine the true epidemiologic characteristics of the pathogen. Suggested measures to help reduce the risk include proper disposal of afterbirth (e.g., deep burial), thorough screening of herdsmen and the animals at the points of entry of animals in the country, including Q fever and other NTDs in the CPD of health professionals, and encouraging farmers to inform physicians of their profession and incidences of abortion on their farms when they visit health centres. This presents a one-health approach to handling Q fever and other zoonoses in Ghana.

## Data availability statement

Data available as supplementary data. Additional data from this study will be made available upon request.

## CRediT authorship contribution statement

**Richard Kwamena Abbiw:** Writing – review & editing, Writing – original draft, Visualization, Methodology, Investigation, Data curation, Conceptualization. **Gloria Ivy Mensah:** Methodology, Formal analysis. **Delphina A.M. Adabie-Gomez:** Writing – review & editing, Supervision, Conceptualization. **Kweku Asare-Dompreh:** Writing – review & editing, Formal analysis. **Stephanie Clement-Owusu:** Methodology. **Vida Yirenkyiwaa Adjei:** Methodology. **Shirley Victoria Simpson:** Methodology. **Mustapha Abubakar Ahmed:** Methodology. **Sherry A.M. Johnson:** Writing – review & editing, Visualization, Supervision, Methodology, Investigation, Conceptualization.

## Declaration of competing interest

The authors declare that they have no known competing financial interests or personal relationships that could have appeared to influence the work reported in this paper.
